# Mechanical and Catalytic Degradation Properties of Porous FeMnCoCr High-Entropy Alloy Structures Fabricated by Selective Laser Melting Additive Manufacturing

**DOI:** 10.3390/ma18010185

**Published:** 2025-01-04

**Authors:** Lyusha Cheng, Cheng Deng, Yushan Huang, Kai Li, Changjun Han

**Affiliations:** 1 College of Mechanical Engineering, ShenZhen Polytechnic University, Shenzhen 518055, China; sherry81@szpu.edu.cn; 2 College of Mechatronics Engineering, Guangdong Polytechnic Normal University, Guangzhou 510635, China; 3 School of Mechanical and Automotive Engineering, South China University of Technology, Guangzhou 510641, China; 202130131139@mail.scut.edu.cn (Y.H.); 202120100662@mail.scut.edu.cn (K.L.)

**Keywords:** additive manufacturing, high-entropy alloys, FeMnCoCr, triply periodic minimal surface, catalytic degradation, laser powder bed fusion

## Abstract

This work investigated the mechanical and catalytic degradation properties of FeMnCoCr-based high-entropy alloys (HEAs) with diverse compositions and porous structures fabricated via selective laser melting (SLM) additive manufacturing for wastewater treatment applications. The effects of Mn content (0, 30 at%, and 50 at%) and topological structures (gyroid, diamond, and sea urchin-inspired shell) on the compression properties and catalytic efficiency of the Fe_80-*x*_Mn*_x_*Co_10_Cr_10_ HEAs were discussed. The results indicated that an increase in the Mn content led to a phase structure transition that optimized mechanical properties and catalytic activities. Among the designed structures, the gyroid HEA structure exhibited the highest compressive yield strength, reaching 197 MPa. Additionally, Fe_30_Mn_50_Co_10_Cr_10_ HEA exhibited exceptional performance in catalytic degradation experiments by effectively degrading simulated pollutants with a significantly enhanced rate by 22.3% compared to other compositions. The Fe_80-*x*_Mn*_x_*Co_10_Cr_10_ HEA catalyst fabricated by SLM demonstrated high stability over multiple cycles. These findings reveal that porous FeMnCoCr-based HEAs have significant potential for catalytic degradation of organic pollutants, providing valuable insights for future catalyst design and development in efficient and sustainable wastewater treatment.

## 1. Introduction

In recent years, the rapid acceleration of global industrialization and urbanization has led to increasingly severe environmental pollution, particularly the contamination of water bodies by organic pollutants. This poses significant threats to both ecosystems and human health. Consequently, it is crucial to develop efficient and environmentally friendly wastewater treatment technologies. Catalytic degradation technology, which involves an effective method for breaking down organic pollutants, has been widely applied in wastewater treatment, especially in peroxymonosulfate (PMS)-activated systems [1]. However, traditional catalysts based on precious metals face limitations due to their high cost and potential secondary pollution despite exhibiting excellent catalytic performance [2]. An increasing part of research focuses on designing and developing non-precious metal-based catalysts that are characterized by high efficiency, stability, and cost-effectiveness [3].

The requirements for catalyst materials include excellent strength and superior catalytic performance. High-entropy alloys (HEAs), compared with their monometallic and bimetallic counterparts, have gained significant attention as promising candidates in the field of catalytic degradation due to their unique compositional design and high-entropy effects, which confer superior mechanical properties and chemical stability. Compared to traditional alloys, HEAs offer unique advantages such as exceptional mechanical properties [4], improved corrosion and oxidation resistance, and enhanced thermal stability [5]. Among them, FeMnCoCr-based HEAs, with extensive compositional tunability, excellent corrosion resistance, and potential catalytic activity, have found wide applications in electrocatalytic oxygen evolution [6], photocatalysis [7], and organic pollutant degradation [8]. Investigations into FeMnCoCr HEAs primarily focus on their phase structures, mechanical properties, and catalytic potential. These alloys exhibit a face-centered cubic (FCC) structure stabilized by varying compositions of Fe, Mn, Co, and Cr. Li et al. investigated the possibility that adjusting the Mn content in FeMnCoCr HEAs plays a crucial role in influencing the stacking fault energy (SFE), thereby affecting the deformation mechanisms and catalytic capabilities of these alloys. For instance, a higher Mn content leads to a transition from a dual-phase structure (FCC and hexagonal close-packed, HCP) to a predominantly FCC phase, enabling optimization of mechanical properties while maintaining high corrosion resistance and catalytic activity.

In addition to material properties, the topological structure also plays a significant role in influencing its catalytic performance. Triply periodic minimal surfaces (TPMS)-based structures have garnered considerable attention in the field of catalytic degradation due to their ability to maximize surface area and pore volume while maintaining mechanical properties. These porous structures not only provide more active sites for catalysis but also increase surface area and material permeability, thus significantly improving catalytic degradation efficiency. Selective laser melting (SLM) additive manufacturing technology offers distinct advantages in fabricating HEAs into porous structures with complex geometries [9,10,11]. Regarding the fabrication of TPMS structures with HEAs using SLM, FeMnCoCr HEAs fabricated using SLM exhibit enhanced structural integrity and catalytic performance [12,13,14]. Junyi Feng et al. fabricated Ti_1.5_NbTa_0.5_ZrMo_0.5_ HEA with a TPMS lattice, utilizing the high cooling rate of SLM to effectively address the issue of elemental segregation and result in refined grains [15]. They obtained a low Young’s modulus, ranging from 6.71 GPa to 16.21 GPa, with excellent compressive properties, achieving near-complete density. Additionally, the average grain size of the SLM-fabricated HEAs was significantly reduced to 5.416 μm compared to its as-cast counterpart (140–200 μm), leading to remarkably enhanced compressive properties and the maintenance of good strength even under extreme deformation, up to 40%.

Despite extensive research on HEAs for catalysis, limitations remain regarding additively manufactured porous HEA structures specifically for wastewater treatment. Therefore, this study aims to investigate the mechanical performance of Fe_80-*x*_Mn*_x_*Co_10_Cr_10_ HEA porous structures with varying compositions fabricated via SLM, including diamond, gyroid, and biomimetic sea urchin-inspired shell topologies, for wastewater treatment. Additionally, the effect of different compositions on the catalytic degradation performance of these HEAs in organic pollutant treatment is explored. By integrating the exceptional mechanical performances of the alloy with the functional characteristics of the structure, diverse wastewater filter support structures are successfully fabricated using SLM. These structures can be utilized for pollutant degradation without requiring any surface treatment, providing new insights for wastewater filtration.

## 2. Materials and Methods

### 2.1. Materials

**Figure 1:**
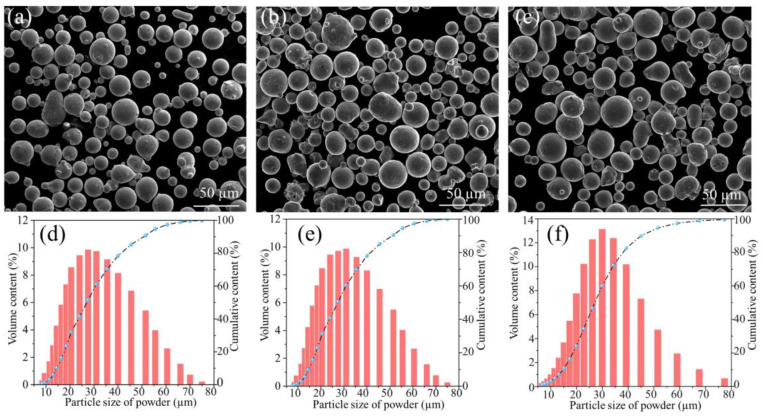
Morphology and particle size distribution of Fe_80-*x*_Mn*_x_*Co_10_Cr_10_HEA powder: (**a**,**d**) Fe_30_Mn_50_Co_10_Cr_10_; (**b**,**e**) Fe_50_Mn_30_Co_10_Cr_10_; (**c**,**f**) Fe_80_Co_10_Cr_10_.

Mn content plays an important role in the phase composition and stability of a FeMnCoCr-based HEA system. Specifically, a Mn content of 40 at% represents a critical point for the transition in deformation mechanisms, shifting from dislocation-dominated plasticity to twinning-induced plasticity and transformation-induced plasticity [16]. Therefore, a broad Mn content range (0–50 at%) was selected for fabrication in this study. Pre-alloyed powders Fe_80-*x*_Mn*_x_*Co_10_Cr_10_ (x = 0, 30 at%, and 50 at%) (Foshan Chengfeng Material Co., Ltd, Foshan, China) were used as the raw materials. The composition elements of different HEA powders are depicted in Table 1. Different powders were prepared by aerosol atomization. Before the processes of SLM forming the alloy and observing the powder morphology image, the powder was placed in a drying oven at 120 °C for 2 h, and the powder morphology is shown in Figure 1a–c. The three powder particles all appeared nearly spherical, and the overall morphology of the powder was excellent. The particle size distribution of the powder is shown in Figure 1d–f. The average particle size of Fe_30_Mn_50_Co_10_Cr_10_, Fe_50_Mn_30_Co_10_Cr_10_, and Fe_80_Co_10_Cr_10_ powders were determined as 28.42 ± 1.42 µm, 30.55 ± 1.53 µm, and 27.01 ± 1.35 µm, respectively.

### 2.2. Porous Fe_80-x_Mn_x_Co_10_Cr_10_ Structures Fabricated by SLM

**Figure 2:**
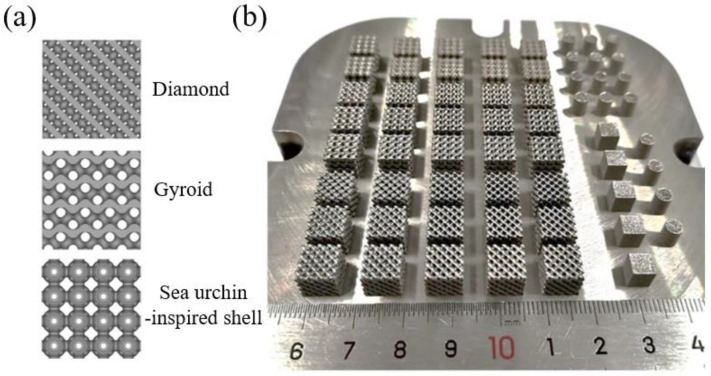
(**a**) Models of diamond, gyroid, and sea urchin-inspired shell structures; (**b**) SLM-fabricated structures using Fe_80-*x*_Mn*_x_*Co_10_Cr_10_ HEAs.

The diamond and gyroid structures are representative of triple-period minimal surface-based structures with the largest specific surface area [17]. Additionally, the sea urchin-inspired shell structure is characterized by a hollow spherical structure with a high pore volume ratio [18]. All three porous structures possess a significant specific surface area, making them suitable for providing catalytic sites. Through the preliminary exploration of the SLM processing of Fe_80-*x*_Mn*_x_*Co_10_Cr_10_ HEAs, the optimal process parameters were selected to fabricate the desired porous structures. Using a laser power of 200 W, scanning speed of 900 mm/s, hatch spacing of 0.07 mm, and powder layer thickness of 0.03 mm [19], porous structures with dimensions of 8 mm × 8 mm × 8 mm were fabricated. Each individual cell within these structures measured 2 mm × 2 mm × 2 mm in size. Furthermore, it should be noted that the diamond and gyroid structures account for 60%, 70%, and 80% of the cells respectively. The sea urchin-inspired shell structure only account for 70% and 80% of cells due to the design limitations. The constraints imposed by the shell structure and small flow apertures limit the achievable porosity in the porous sea urchin structures, preventing designs with porosities higher than 60%. Figure 2b illustrates various compositions of SLM-fabricated porous structures made from Fe_80-*x*_Mn*_x_*Co_10_Cr_10_ HEA alongside bulk and cylindrical compression samples.

### 2.3. Catalytic Degradation

The fabricated Fe_80-*x*_Mn*_x_*Co_10_Cr_10_ HEA porous structures were employed for the activation of PMS to catalyze the degradation of organic wastewater. To demonstrate the superiority of the HEAs in activating PMS, a control test was conducted using a Fenton system under identical conditions. A simulated pollutant wastewater containing acid red G (ARG) solution with a concentration of 10 mg/L was used. Moreover, different components of Fe_80-*x*_Mn*_x_*Co_10_Cr_10_ catalyst HEAs and a PMS solution with a concentration of 0.5 g/L or 20 mM HO solution were added to the pollutant solution in various experimental groups. At regular intervals during the catalytic test, the concentration of organic matter in the solution was measured using UV-1900PC ultraviolet visible spectrophotometry produced by the Shanghai Aoyi Instrument Company (Shanghai, China), of which the wavelength range is 190–1100 nm and the accuracy range is ±0.3 nm. The degradation rate was determined by calculating the difference between the initial concentration and the concentration at specific time points, which was then compared with the initial solution concentration to evaluate catalytic degradation efficiency. The elemental valence changes before and after the catalysis of high-entropy alloys were tested using an Axis Supra+ photoelectron spectrometer (XPS) produced by the British company Kratos (Manchester, British). The alloy test surface was polished with SiC sandpaper until it was smooth and flat to remove oil stains and powder impurities on the sample surface, and the valence changes before and after the catalysis of the elements were analyzed.

To prove that the catalyst HEAs exhibited universal applicability, rhodamine B (RhB) and tetracycline hydrochloride (TC) solutions were also used as organic pollutants for degradation tests under conditions identical to ARG. After each catalytic degradation test, both the HEA catalyst and its degraded alloy were cleaned and dried using anhydrous ethanol to investigate their catalytic degradation effect; this process was reciprocated multiple times to assess cyclic stability in terms of catalytic degradation efficiency for materials. Additionally, the influence on the catalytic degradation system was explored by manipulating the initial pH of the solution and introducing anions into the solution. Different types of free radical-trapping agents were employed to study the active free radicals in the Fe_80-*x*_Mn*_x_*Co_10_Cr_10_/PMS reaction system. Finally, the catalytic performance of different porosities and types of HEA porous structures were examined, which laid a foundation for further validation of the feasibility of the fabricated porous catalytic degradation reactors.

### 2.4. Characterizations and Mechanical Testing

To observe the surface morphology of the fabricated HEAs and their porous structures, a Zeiss Axiolab5 microscope from Carl Zeiss AG (Oberkochen, Germany) was used. The microscopic characterization of the polished alloy surface morphology was conducted using Optical microscopy a NovaNanoSEM430 scanning electron microscope (SEM). The polished surface was etched with aqua regia (HNO_3_: HCl = 1:3) for 30 s, followed by extensive rinsing with alcohol and water to prepare it for the observation of microstructure defects. The prepared alloy was used for tests to observe microstructure features such as grain size, orientation, and phase composition. The tensile fracture alloy was ultrasonically cleaned in acetone to remove residual impurities and grease, enabling the observation of the fracture microstructure.

The compression tests were conducted using the CMT5105 electronic universal testing machine from the Zhuhai Sansitaijie Corporation (Zhuhai, China). The porous compression structures included gyroid, diamond, and sea urchin-inspired shell structures, with a loading rate of 0.5 mm/min during the compression tests. Three samples were tested to determine the compression properties of the HEA structures.

## 3. Results

### 3.1. Relative Density

**Figure 3:**
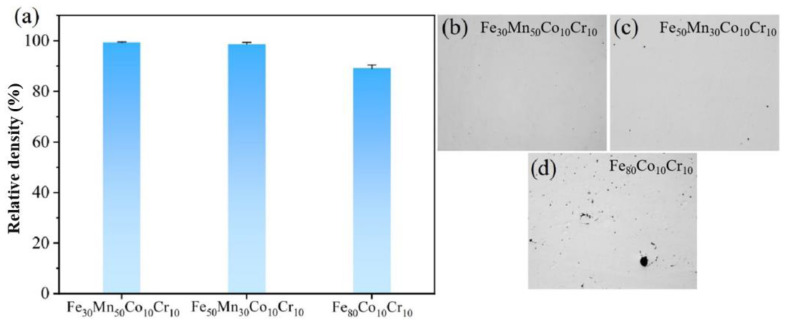
(**a**) Relative density of SLM-fabricated HEAs; (**b**–**d**) surface morphologies of Fe_30_Mn_50_Co_10_Cr_10_, Fe_50_Mn_30_Co_10_Cr_10_, and Fe_80_Co_10_Cr_10_, respectively.

The relative densities of three Fe_80-*x*_Mn*_x_*Co_10_Cr_10_ HEA samples were investigated, as shown in Figure 3. The results indicated that the relative densities of Fe_30_Mn_50_Co_10_Cr_10_, Fe_50_Mn_30_Co_10_Cr_10_, and Fe_80_Co_10_Cr_10_ HEA were measured as 97.8%, 96.5%, and 94.3%, respectively, indicating the influence of Mn content on the relative densities of the HEAs. A higher Mn content was correlated with a slight improvement in density values. The SEM images revealed that while the surfaces of the Fe_30_Mn_50_Co_10_Cr_10_ and Fe_50_Mn_30_Co_10_Cr_10_ HEAs appeared smooth without any obvious defects, the Fe_80_Co_10_Cr_10_ HEA displayed a few visible voids, which was consistent with its lower relative density.

### 3.2. Morphology of the HEA Structures

**Figure 4:**
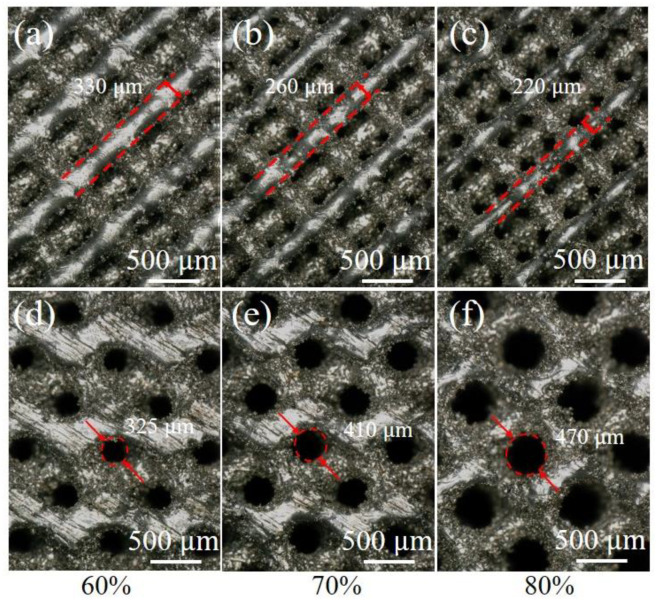
Optical morphologies of Fe_30_Mn_50_Co_10_Cr_10_ HEA structures with different porosities: (**a**–**c**) diamond structure; (**d**–**f**) gyroid structure.

Figure 4 illustrates the optical morphology of the diamond and gyroid porous Fe_30_Mn_50_Co_10_Cr_10_ structures. It can be observed that an increase in porosity from 60% to 80% led to a rise in pore diameter for the gyroid structure, expanding from 325 μm to 470 μm. This increase contributed to a reduction in pillar diameter for the diamond structure, decreasing from 330 μm to 220 μm. Within these three distinct pores, powder particles forming the struts during SLM exhibited significant adhesion phenomena. This can be attributed to the layered superposition effect caused by the laser scanning trajectory on the curved struts. When the laser melted each powder layer, heat penetrated into subsequent solidified layers and radiated towards the powder at the edges of laser scanning trajectories, causing them to melt and adhere to boundaries between the melted layers on the curved struts.

**Figure 5:**
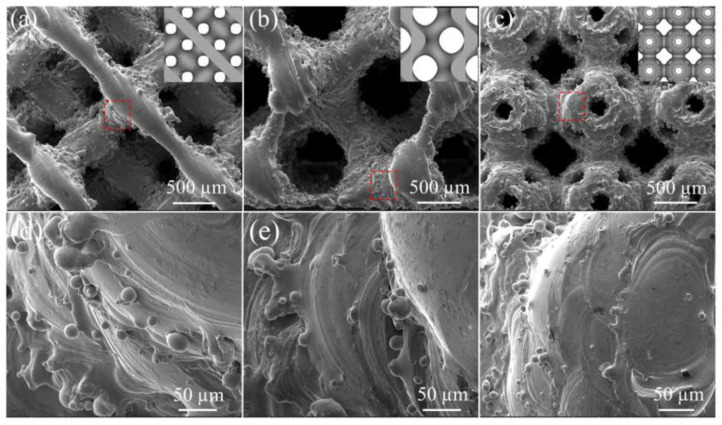
SEM morphology of different Fe_30_Mn_50_Co_10_Cr_10_ structures with 80% porosity: (**a**,**d**) diamond structure; (**b**,**e**) gyroid structure; (**c**,**f**) sea urchin-inspired shell structure.

The SEM micromorphology of the three HEA structures with a porosity of 80% is presented in Figure 5. The fabricated HEA structures exhibited step-like surfaces, with powder particles adhering to their edges. This phenomenon can be attributed to the layer-by-layer melting of powder during the fabrication of the structures, characterized by curved and smooth connection between unicellular crystals. Due to the Gaussian heat source generated by the laser spot, deviations in the laser scanning track along the curved surface result in accumulated heat and penetration effects, causing nearby or upper-layer powder particles to melt and adhere to the struts.

### 3.3. Compression Properties

**Figure 6:**
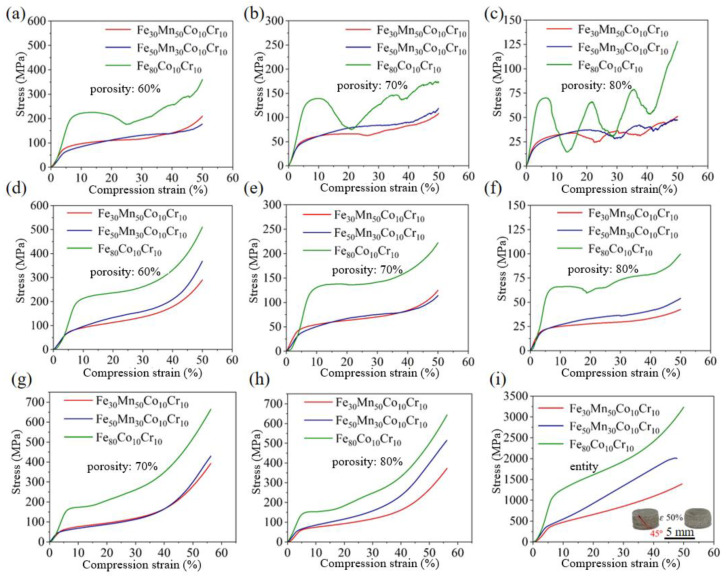
Stress–strain curves of Fe_80-*x*_Mn*_x_*Co_10_Cr_10_HEA structures: (**a**–**c**) diamond; (**d**–**f**) gyroid; (**g**,**h**) sea urchin-inspired shell structure; (**i**) cylindrical bulk.

Figure 6 depicts the compressive stress–strain curves of the three types of Fe_80-*x*_Mn*_x_*Co_10_Cr_10_ structures with various compositions, and their specific compressive yield strengths are outlined in Table 2. The compression process can be delineated into three distinct stages: the linear elastic deformation stage, the yield platform stress stage, and the densification stage. Notably, the initial segment of the curve during the elastic stage exhibited nonlinearity and concavity, which could be potentially be attributed to the surface roughness and size deviation of the fabricated structures and uneven loading distribution across unit cells, as well as discrepancies in the equipment joint alignment. After a minor strain, there was a rapid increase in stress, leading to a peak value. Subsequently, slight fluctuations in stress occurred along with strain due to lattice strut collapse, resulting in an extended yield platform stress stage. Ultimately, complete collapse of the structures caused a sharp increase in stress and subsequent densification. When compared to the gyroid and diamond structures, the latter exhibited a higher susceptibility to collapse under compressive forces. During the compression of Fe_80_Co_10_Cr_10_ diamond structure, the stress fluctuated within the yield platform stress stage, and then rapidly decreased once it reached its peak value. This behavior was ascribed to the sequential failure of lattice layers within the porous structures; as load was transferred from top to bottom, lower lattices failed prematurely before upper lattice collapse after compaction. The load-bearing limit was reached by the lower lattice before reaching that of the upper lattice. Moreover, this phenomenon occurred progressively in stages as the porosity increased from 60% to 80%. Conversely, Fe_30_Mn_50_Co_10_Cr_10_ and Fe_50_Mn_30_Co_10_Cr_10_ HEA structures exhibited similar fluctuations at 80% porosity when using a diamond structure. Across different compositions, all Fe_80-*x*_Mn*_x_*Co_10_Cr_10_ HEAs demonstrated stable compression processes within the gyroid structure. Only at 80% porosity did slight stress fluctuations occur in the Fe_80_Co_10_Cr_10_ HEA; however, they quickly recovered back to normal levels. The compression properties of the gyroid structure were better than the diamond structure for all the HEAs. In SLM, defects like unfused powder, cracks, and porosity affect alloy compressive properties by causing stress concentration and reducing strength and ductility. However, structure impact outweighs defect impact due to consistent fabrication parameters.

Figure 6g,h display the compressive stress–strain curves of the sea urchin-inspired shell structures made from Fe_80-*x*_Mn*_x_*Co_10_Cr_10_ HEAs with various compositions. The specific compressive yield strengths of the structures are provided in Table 2. Notably, this structure exhibited superior compression performance compared to both diamond and gyroid structures. At a porosity level of 80%, no stress fluctuation was observed in the yield platform stage, highlighting the robust stability of the sea urchin-inspired shell structures. Additionally, Figure 6i compares the compressive stress–strain curves of SLM-fabricated HEA bulks to those of porous structures. It is worth noting that at a strain of 50% in the Fe_50_Mn_30_Co_10_Cr_10_HEA, the fracture path of the compressed structure diverged at a 45° angle from the direction of the applied load, resulting in shear failure. This failure mode can be attributed to the martensitic transformation occurring during deformation in FeMnCoCr-based HEAs [20]. The stress incompatibility between the martensitic phase and austenitic phase matrix made the alloy susceptible to crack formation and pore generation during compression. It is speculated that while martensitic transformation enhanced the mechanical strength of Fe_50_Mn_30_Co_10_Cr_10_ HEA during compression, it also compromised its ductility [21].

In comparison to the Fe_30_Mn_50_Co_10_Cr_10_ and Fe_50_Mn_30_Co_10_Cr_10_ HEAs, the Fe_80_Co_10_Cr_10_ HEA demonstrated significantly superior compression properties than the former two alloys, while the performance of Fe_50_Mn_30_Co_10_Cr_10_ alloy was comparatively inferior. As the Mn content increased from 0 to 50%, there was a gradual decrease in compressive strength within all the porous structures. This can be attributed to a phase structure transformation from BCC to HCP + FCC and finally FCC [22,23], resulting in significant differences in microstructure that influence the compressive properties of different HEAs [24]. Both diamond and gyroid structures exhibited decreased compressive properties with decreasing porosity; among them, the diamond structure displayed relatively poorer performance compared to the sea urchin-inspired shell structure, which demonstrated superior performance. Within the same structural configuration, higher structural porosity correlated with lower yield strength and flow stress. This phenomenon can be attributed to the reduced strut sizes caused by increased porosity, which induces greater stress concentrations under equivalent loading conditions, thereby decreasing the mechanical properties of the structures. Therefore, when designing and manufacturing porous HEA structures, a thorough understanding of how porosity gradients influence stress distribution is crucial for ensuring the safety and reliability of these structures. The yield strength of porous structures decreases with increasing porosity, as evidenced by the significant drops observed in diamond and gyroid structures between 60% and 80% porosity. Despite its superior compression performance compared to both diamond and gyroid structures, the sea urchin-inspired shell structure also follows this trend. Although porous materials offer advantages such as a large surface area and good permeability, which are beneficial for catalytic applications, their mechanical strength is notably lower than that of bulk materials. Therefore, it is essential to balance these properties against the intended application and expected mechanical loads.

### 3.4. Catalytic Properties

**Figure 7:**
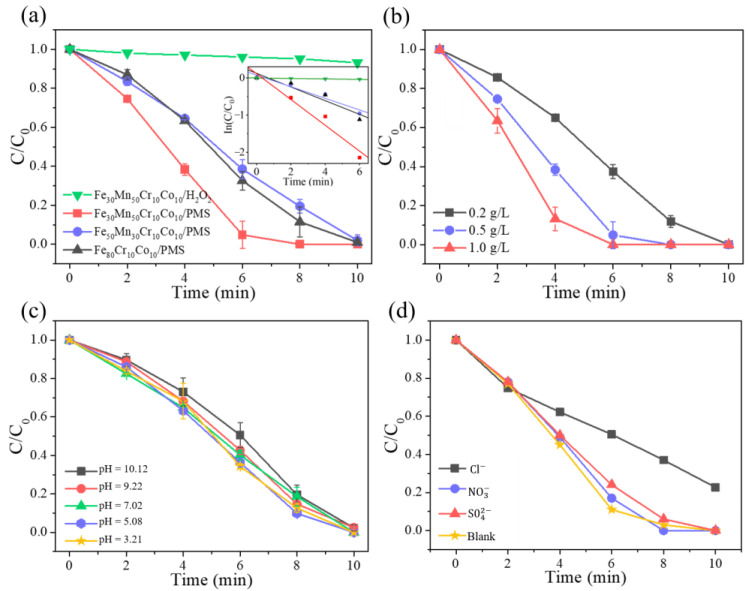
(**a**) Degradation of ARG by different catalysts and different reaction systems; (**b**) degradation of ARG by different concentrations of PMS reaction system; (**c**) effect of different initial pH on the degradation of ARG; (**d**) effects of co-existing anions on ARG degradation.

Figure 7a presents the comparison of degradation effect of Fe_80-*x*_Mn*_x_*Co_10_Cr_10_ HEA across diverse catalysts and reaction systems, especially in a 0.5 g/L PMS solution and a 20 mM HO solution. A pseudo-first-order kinetics analysis for calculating the reaction rates was used. The relevant statistical data are presented as follows: 0.0065 min^−1^ for Fe_30_Mn_50_Cr_10_Co_10_/H_2_O_2_, 0.34643 min^−1^ for Fe_30_Mn_50_Cr_10_Co_10_/PMS, 0.15609 min^−1^ for Fe_50_Mn_30_Cr_10_Co_10_/PMS, and 0.18378 min^−1^ for Fe_80_Cr_10_Co_10_/PMS. Notably, the Fe_30_Mn_50_Co_10_Cr_10_/PMS system exhibited remarkable performance by completely degrading ARG within 8 min. This system demonstrated significantly superior catalytic rates and efficiencies compared to the Fe_50_Mn_30_Co_10_Cr_10_/PMS and Fe_80_Co_10_Cr_10_/PMS systems under identical conditions. Conversely, in the Fe_30_Mn_50_Co_10_Cr_10_/H_2_O_2_ system, the ARG pollutant solution could hardly be degraded. The results indicated that the degradation effect of the bulk alloy on ARG varied among different systems, with Fe_50_Mn_30_Co_10_Cr_10_/PMS being superior to both the Fe_30_Mn_50_Co_10_Cr_10_/PMS and Fe_80_Co_10_Cr_10_/PMS systems. It also outperformed the Fe_30_Mn_50_Co_10_Cr_10_/H_2_O_2_ system. With increasing the Mn content, the catalytic activity of the HEA was enhanced, leading to selecting Fe_30_Mn_50_Co_10_Cr_10_ HEA as the main catalyst for further research. The exploration of the optimal oxidant dosage for the Fe_30_Mn_50_Co_10_Cr_10_/PMS system is further illustrated in Figure 7b. At 4 min, with PMS concentrations of 0.2 g/L, 0.5 g/L, and 1 g/L, the ARG degradation rates were determined to be 35%, 61.7%, and 86.8%, respectively. Complete degradation of ARG was achieved within a time frame of only 6 min when the PMS concentration reached 1 g/L. Similarly, at a PMS concentration of 0.5 g/L, an impressive degradation rate of ARG amounting to 95.2% was observed within just 8 min. Furthermore, even at a lower PMS concentration of only 0.2 g/L, significant degradation progress was evident with an ARG degradation rate reaching up to 62.6%. When the concentration of PMS was 0.5 g/L, a significant enhancement in the degradation efficiency of ARG was improved. However, upon increasing the PMS concentration to 1 g/L, no further improvement in the degradation efficiency of the system was observed. Considering both economic and environmental factors, an optimal dosage of PMS at 0.5 g/L was determined.

The catalytic degradation effect of the Fe_30_Mn_50_Co_10_Cr_10_/PMS system on ARG was investigated under different solution pH levels, as depicted in Figure 7c. It can be observed that the trend of ARG degradation efficiency remained consistent across various initial solution pH values. Compared to alkaline conditions, a slightly higher catalytic degradation efficiency was achieved when the initial solution was acidic for the Fe_30_Mn_50_Co_10_Cr_10_/PMS system. Within 6 min, at a pH of 3.21, the catalytic degradation rate of ARG reached 65.7%, while it decreased to 49.5% at a pH of 10.12. After 10 min of catalytic degradation, ARG exhibited a degradation rate of 97.6% across different pH levels, indicating that the Fe_30_Mn_50_Co_10_Cr_10_/PMS system effectively degraded simulated pollutants over a wide range of pH (3.21–10.12). However, it should be noted that in real pollutant wastewater samples, the presence of coexisting ions such as ${Cl}^{-}$, ${NO}_{3}^{-}$, and ${SO}_{4}^{2-}$ may influence the degradation performance of the Fe_30_Mn_50_Co_10_Cr_10_/PMS system. The degradation effect of different anions in the Fe_30_Mn_50_Co_10_Cr_10_/PMS system is illustrated in Figure 7d. In order to conduct the catalytic degradation test, solutions containing 1 mg/L NaCl, NaNO_3_, and Na_2_SO_4_ were added to the initial solution of the Fe_30_Mn_50_Co_10_Cr_10_/PMS system. As depicted in the figure, ${NO}_{3}^{-}$ and ${SO}_{4}^{2-}$ did not exert any influence on the degradation performance of ARG pollutants in the Fe_30_Mn_50_Co_10_Cr_10_/PMS system, with a removal rate reaching 100% within 10 min. Conversely, ${Cl}^{-}$ significantly inhibited the degradation performance of the Fe_30_Mn_50_Co_10_Cr_10_/PMS system, resulting in a decrease of ARG degradation rate to 77.4% within 10 min. This can be attributed to ${Cl}^{-}$ reacting with HO· and ${SO}_{4}^{-}$ in solution to form Cl·, both Cl· and ${Cl}^{-}$ exhibited high reaction rates and readily formed ${Cl}_{2}^{-}$, which possessed relatively weak oxidation ability. Consequently, both the degradation rate and efficiency of ARG in this system were reduced. Therefore, an inhibition effect was observed when ${Cl}^{-}$ co-existed.

**Figure 8:**
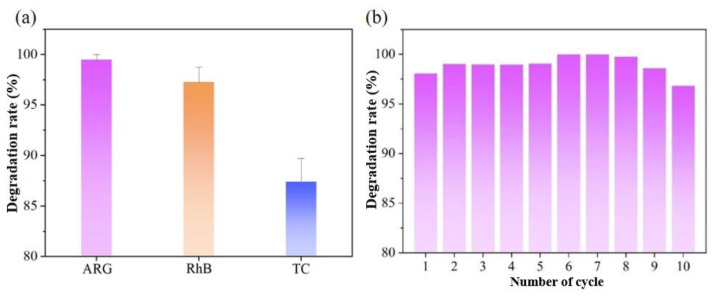
(**a**) Universality of catalyst for degradation of different organic pollutants; (**b**) cycle stability of catalyst for degradation of ARG.

To demonstrate the universal applicability of the Fe_30_Mn_50_Co_10_Cr_10_ catalyst HEA, two additional organic pollutants, RhB and TC, were employed at a concentration of 10 mg/L for the pollutant reagent and 0.5 g/L for the oxidant PMS. This experiment was carried out under identical test conditions as those used for ARG degradation to explore the effect of the Fe_30_Mn_50_Co_10_Cr_10_ catalyst on diverse pollutants. Figure 8a illustrates that the Fe_30_Mn_50_Co_10_Cr_10_/PMS system catalyzed the degradation of different organic substances at various rates, demonstrating its ability to maintain a high degradation rate across different pollutants. Within 10 min, nearly complete degradation was achieved for ARG, while RhB achieved a degradation rate of 98.7% and TC reached a rate of 87.4%. These results confirm that the Fe_30_Mn_50_Co_10_Cr_10_/PMS system is capable of degrading a range of pollutants with excellent universal applicability. As mentioned above, the Fe_30_Mn_50_Co_10_Cr_10_ bulk catalyst effectively activated persulfate PMS to catalyze the degradation of simulated organic pollutants, showcasing a high degradation efficiency that remained largely unaffected by the coexistence of ions in the degraded solution. This catalyst exhibited remarkable adaptability for catalytic degradation under various acid-based environmental conditions. However, further research is required to investigate the stability of the Fe_30_Mn_50_Co_10_Cr_10_ catalyst HEAs for their widespread application in wastewater treatment. Figure 8b depicts the catalytic effect of Fe_30_Mn_50_Co_10_Cr_10_ catalyst HEAs after 10 cycles of usage. It is evident that even after 10 cycles of catalytic degradation using the Fe_30_Mn_50_Co_10_Cr_10_ bulk, the catalytic degradation rate of ARG by the Fe_30_Mn_50_Co_10_Cr_10_/PMS system could still remain above 96.4%, indicating an excellent stable catalytic degradation effect.

**Figure 9:**
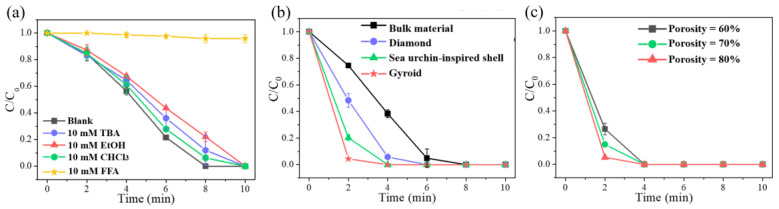
(**a**) Effects of different trapping agents on ARG degradation, and C/C0 indicates the ARG degradation rate; (**b**) different structural types of Fe_30_Mn_50_Co_10_Cr_10_; (**c**) different porosity of Fe_30_Mn_50_Co_10_Cr_10_ gyroid structure.

To investigate the active groups that played a significant role in the Fe_30_Mn_50_Co_10_Cr_10_/PMS system, free fatty acid (FFA), CHCl_3_, ethanol (EtOH), and tert-butanol (TBA) were employed as free radical-trapping agents at a concentration of 10 mM each to capture these active groups. During the catalytic experiment, at regular intervals, the concentration of organics in the solution was tested using a UV-visible spectrophotometer. The degradation rate of catalytic degradation was obtained by comparing the difference, C, between the initial concentration, C_0_, and the concentration, C_t_, measured at a certain time point, with the initial solution concentration, C_0_. As seen in Figure 9a, the introduction of EtOH and TBA to the solution led to noticeable suppression of ARG degradation in the Fe_30_Mn_50_Co_10_Cr_10_/PMS system, indicating the generation of HO· and ${SO}_{4}^{-}$. The inclusion of CHCl_3_ reduced the degradation rate slightly due to its ability to capture O_2_^−^, which hindered its conversion into ^1^O_2_. Notably, FFA significantly inhibited ARG degradation and almost completely halted the reaction with a decrease in degradation rate by 4.3% within 10 min, suggesting that ^1^O_2_ was indeed the primary active group present.

Building upon this experimental foundation regarding ARG degradation by Fe_30_Mn_50_Co_10_Cr_10_ bulk in PMS reaction systems, the effects of diamond, gyroid, and sea urchin-inspired shell structures on the ARG pollutant degradation in heterogeneous activated PMS reaction systems were further investigated. The degradation effects of ARG were compared between the HEA bulk and its diverse porous structures in the PMS reaction system, as shown in Figure 9b. Within 2 min, the gyroid structure exhibited a remarkable degradation rate of over 95.7% for ARG pollutants, followed by the sea urchin-inspired shell structure with a rate of 79.8%. Additionally, the diamond structure achieved a degradation rate of 51.7%, while the HEA bulk reached only 25.4%. These results indicated that both catalytic efficiency and degradation rate were significantly higher for the gyroid structure compared to other structures. Both the gyroid and sea urchin-inspired shell structures demonstrated complete degradation of ARG within 4 min, whereas it took the diamond structure 6 min to achieve complete removal of ARG pollutants. On the other hand, the bulk HEA degraded within 8 min. It is hypothesized that the abundance of reactive sites on its surface contributed to higher catalytic efficiency in case of the gyroid HEA structure. To explore further impact on catalytic degradation effect due to different porosities, the gyroid structure using Fe_30_Mn_50_Co_10_Cr_10_ HEA was selected (Figure 9c). Increasing porosity from 60% to 80% resulted in corresponding increase in degradation rates for ARG within the first 2 min were 73.5%, 85.1% and 94.7%, respectively. All structures with three porosities completely degraded ARG pollutants within 4 min. Larger porosity facilitated more catalytic sites and larger specific surface area, enabling full utilization of functional catalytic properties without compromising mechanical strength.

**Figure 10:**
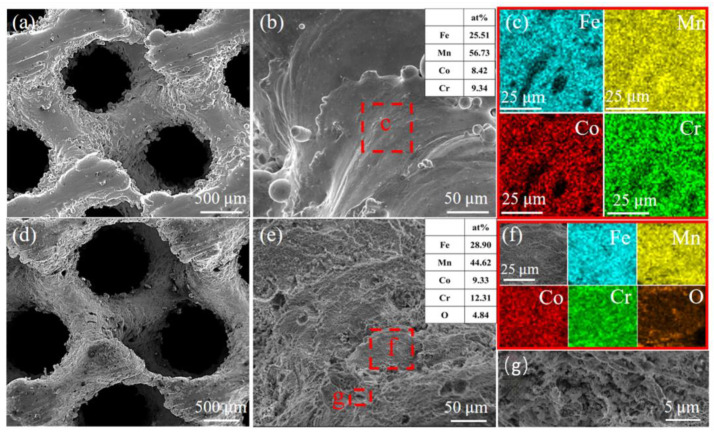
Micromorphology of Fe_30_Mn_50_Co_10_Cr_10_ gyroid structure and EDS analysis: (**a**–**c**) before catalytic degradation; (**d**–**g**) after catalytic degradation.

For the gyroid HEA structure with 80% porosity, the microscopic morphology and composition energy spectrum analysis were conducted both before and after undergoing catalytic degradation. Prior to catalysis, as depicted in Figure 10a–c, the surface exhibited noticeable adhesion of powder particles and featured a smooth morphology with distinct stepped texture. The slicing data of the gyroid structure model was output in differential layers, which led to the formation of a striped step. The surface energy spectrum analysis revealed the accumulation of Mn at the edge of each step, due to rapid laser deflection and scanning along this region where Mn diffusion and evaporation occurred more readily. The lowest saturated vapor pressure of Mn resulted in a strong Marangoni effect within the molten pool, facilitating its enrichment at the edge. After being catalyzed by the gyroid structure (Figure 10d–g), significant changes were observed on the surface, including loss of smoothness and unclear platform layer. Instead, a large number of pits and layered folds appeared as a result of reaction between metal elements on the surface and PMS oxidant, promoting surface corrosion to form pits. The presence of O element on the surface after catalytic degradation further verified the activation of PMS system of the Fe_30_Mn_50_Co_10_Cr_10_ HEA, generating ^1^O_2_ for ARG pollutant degradation.

**Figure 11:**
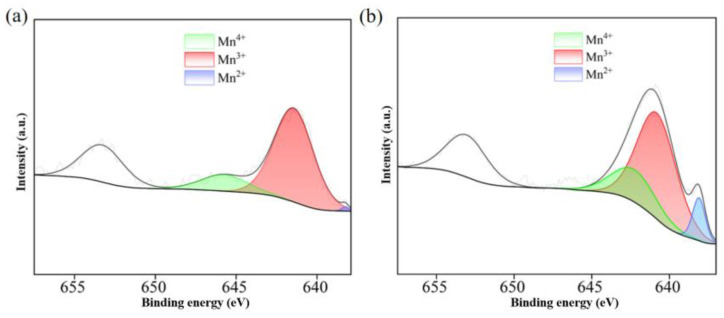
XPS spectra of Mn 2p for Fe_30_Mn_50_Co_10_Cr_10_ HEA: (**a**) after catalysis; (**b**) before catalysis.

The catalytic degradation tests were carried out to investigate the efficiency, stability, and universality of Fe_80-*x*_Mn*_x_*Co_10_Cr_10_ HEA catalysts. Additionally, capture tests and characterization analysis were proposed to study the potential mechanism of ARG degradation by the Fe_30_Mn_50_Co_10_Cr_10_/PMS system. During the heterogeneous activation process of PMS using the Fe_30_Mn_50_Co_10_Cr_10_ catalyst, a REDOX reaction is essentially involved. PMS itself exhibits strong oxidation properties, facilitating electron transfer from the HEA surface and leading to the breaking of molecular valence bonds in PMS oxidants. Consequently, free groups with high oxidation properties are formed. This property makes PMS suitable for organic matter oxidation in wastewater treatment processes. Due to the strong oxidative nature of PMS, electrons are lost from the HEA surface, acting as a reducing agent while transforming the valence state of HEAs from low to high states [25,26] (Formulas (1)–(3)). The valence of Mn before and after catalysis was characterized using X-ray photoelectron spectroscopy (XPS) (Figure 11). The Mn 2p3/2 spectrum exhibited three different peaks, with binding energies of 638.2 eV for Mn^2+^, 641.5 eV for Mn^3+^, and 645.9 eV for Mn^4+^ (Figure 11a), resulting in energy differences between different valence states of 3.3 eV and 4.4 eV, respectively. However, a significant difference in the binding energy of Mn^4+^ was observed before and after the alloy-catalyzed reaction, with a value of approximately 642.7 eV (Figure 11b). This discrepancy can be attributed to the promotion of abundant oxygen vacancies through the conversion from Mn^4+^, which demonstrates remarkable redox properties during PMS activation processes. In addition, the deviation between binding energies determined the degree of conversion among different valence elements [27], where small differences are more likely to undergo conversion reactions. The mutual conversions among Mn^2+^, Mn^3+^, and Mn^4+^ played an important role in activating PMS reactions. The conversion from M^n+^ to M^(n+1)+^ ions (where n = I, II, III, IV) is more likely to occur than their corresponding reverse reactions. The above analysis further supports that within the PMS reaction system, there exists a process whereby low-priced metal species (M^0^) are converted into high-priced metal ions (M^n+^, M^(n+1)+^).

M^0^ → M^n+^ + ne^−^ (1)

(2) $$M^{n+}+{HSO}_{5}^{-}\to M^{(n+1)+}+{SO}_{4}^{-}+{OH}^{-}$$

(3) $$M^{(n+1)+}+{HSO}_{5}^{-}\to {Mn}^{+}+{SO}_{5}^{-}+H^{+}$$

By introducing FFA to the reaction solution, it was observed that the catalytic reactive group in the Fe_30_Mn_50_Co_10_Cr_10_/PMS system is ^1^O_2_, which could be attributed to a REDOX reaction resembling metal–SO_5_^-^ (Formulas (4) and (5)) interaction, leading to the generation of metal ions with higher valence states. Mn is able to play a dual role as both a reducing and oxidizing agent through a synergistic mechanism [28]. Specifically, Mn (III)/Mn (IV) undergoes dynamic cyclic transformation (Formulas (6)–(8)), where Mn (III) acts as a reducing agent by losing electrons and forms complexes with absorbed PMS to generate Mn (IV). Furthermore, Mn (IV), functioning as an oxidizing agent, participates in electron transfer from PMS during ARG degradation and generates intermediate compounds such as H_2_O and CO_2_ (Formula (9)). This repetitive cycle greatly improves PMS activity and accelerates organic pollutant degradation. In addition, excessive accumulation of Mn (II) content hinders further REDOX reaction, which is supported by the valence mapping of Mn element shown in Figure 11.

(4) Mn++SO5−→M(n+1)++SO42−+0.5O21

^1^O_2_ + ARG → intermediate + H_2_O + CO_2_ (5)

(6) $$Mn(III)+{{HSO}_{5}}^{-}\to Mn(III)-{\left(HO\right)OSO}_{3}^{-}$$

(7) $$Mn\left(III\right)-{\left(HO\right)OSO}_{3}^{-}+H^{+}\to Mn(IV)+{SO}_{4}^{2-}+H_{2}O+e^{-}$$

Mn(IV) + e^−^ → Mn(III) (8)

Mn(IV)/Mn(III) + ARG → intermediate + H_2_O + CO_2_ (9)

**Figure 12:**
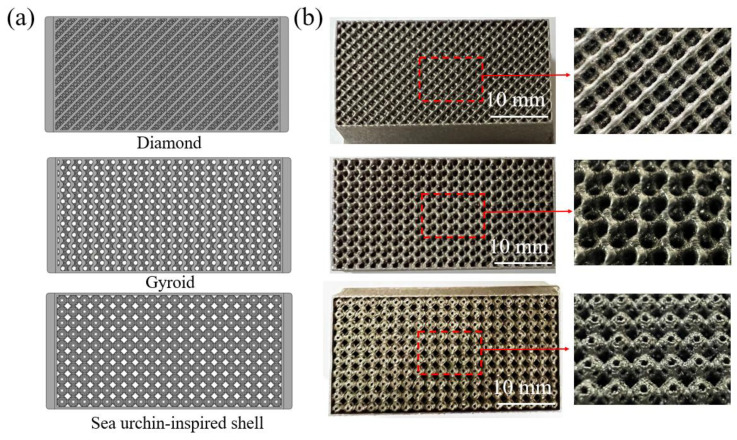
Fabrication of Fe_30_Mn_50_Co_10_Cr_10_ catalytic degradation filters with three porous structures via SLM: (**a**) model diagrams of different porous structures; (**b**) images of the fabricated structures and their structural morphologies.

In summary, the unique compositional design and high-entropy effects of Fe_80-*x*_Mn*_x_*Co_10_Cr_10_ HEAs enable these alloys to exhibit stable and efficient catalytic degradation performance, demonstrating their potential as key components in wastewater purification systems. To verify the industrial applicability of this concept, Fe_30_Mn_50_Co_10_Cr_10_ HEA was employed to fabricate filters with three distinct porous structures (Figure 12). These filters exhibited no macroscopic defects such as warping or cracking, measuring 40 mm × 20 mm × 14 mm with a single cell lattice 2 mm in size, specifically designed to enhance the decomposition of organic pollutants during the wastewater filtration process.

## 4. Conclusions

In this study, FeMnCoCr HEAs with diverse compositions and porous structures were fabricated using SLM, and their catalytic degradation capabilities towards organic pollutants were systematically investigated. The effects of Mn content and topological structures on the compression properties and catalytic efficiency of the Fe_80-*x*_Mn*_x_*Co_10_Cr_10_ HEAs were discussed.

An increase in Mn content led to a phase structure transition from a dual-phase (FCC and HCP) composition to a predominantly FCC phase composition, thereby optimizing the mechanical properties and catalytic activities of the HEAs. The relative densities of Fe_30_Mn_50_Co_10_Cr_10_, Fe_50_Mn_30_Co_10_Cr_10_, and Fe_80_Co_10_Cr_10_ HEAs were determined to be 97.8%, 96.5%, and 94.3%, respectively.

The gyroid structure exhibited stable compressive stress–strain curves with minimal stress fluctuations, whereas the diamond structure demonstrated a tendency to collapse after reaching peak stress. Additionally, the biomimetic sea urchin-inspired shell structure surpassed other structures in mechanical properties, especially at 80% porosity. The compressive yield strength of the Fe_30_Mn_50_Co_10_Cr_10_ alloy varied significantly across different structures, with the gyroid structure boasting the highest yield strength at 197 MPa, in contrast to the diamond structure that displayed the lowest yield strength at merely 60 MPa. In catalytic degradation experiments, the Fe_30_Mn_50_Co_10_Cr_10_ HEA exhibited exceptional organic pollutant degradation performance in a PMS system, effectively degrading simulated pollutants such as Acid Red G, RhB, and TC. Compared to Fe_50_Mn_30_Co_10_Cr_10_ and Fe_80_Co_10_Cr_10_ HEAs, Fe_30_Mn_50_Co_10_Cr_10_ exhibited a significantly enhanced catalytic degradation rate by 22.3%, achieving a remarkable 97.6% degradation rate of Acid Red G within just 8 min.

The catalyst demonstrated high stability over multiple cycles, maintaining a degradation rate above 96.4% after ten cycles. Furthermore, the different types of porous structure significantly affected the catalytic degradation performance. In particular, the gyroid structure achieved a degradation rate exceeding 95.7% within 4 min, while the diamond structure and bulk metal achieved degradation rates of 51.7% and 25.4%, respectively, confirming the advantages of porous structures in enhancing catalytic performance.

Porous FeMnCoCr HEA structures fabricated through SLM demonstrated significant potential for catalytic degradation of organic pollutants. These HEA structures not only exhibited superior mechanical properties and structural stability but also possessed high catalytic activity and wide applicability. During the manufacture of porous HEA parts using SLM, it is crucial to address the potential challenges associated with scaling up the process. These challenges include maintaining consistent material quality, enhancing process efficiency, and overcoming equipment limitations. Fabricating complex structures such as gyroid, sea urchin-inspired shell structures, and diamond structures through this process holds significant importance. These HEA structures, characterized by their unique physical resilience and chemical stability, exhibit great potential for applications in fields such as mechanics and catalysis. Thus, conducting comprehensive research on the SLM processing of these structures and addressing the challenges associated with their large-scale production are essential for advancing the widespread adoption of HEAs across various industries. This study presents a novel perspective on efficient and sustainable wastewater treatment technologies, offering valuable insights for future catalyst design and development.

## Figures and Tables

**Table 1:** Types and component elements of Fe_80-*x*_ Mn*_x_*Co*_x_*Cr_10_ HEA powders.

Pre-Alloyed Powder (wt%)	Fe	Mn	Co	Cr
Fe_30_Mn_50_Co_10_Cr_10_	30.6	49.87	10.22	9.31
Fe_50_Mn_30_Co_10_Cr_10_	50.75	29.38	10.42	9.45
Fe_80_Co_10_Cr_10_	80.12	-	10.56	9.32

**Table 2:** Yield strength of the SLM-fabricated Fe_80-*x*_Mn*_x_*Co_10_Cr_10_HEA structures.

Unit Cell	Porosity	Alloy Composition	Yield Strength (MPa)
Diamond	60%	Fe_30_Mn_50_Co_10_Cr_10_	60
		Fe_50_Mn_30_Co_10_Cr_10_	47
		Fe_80_Co_10_Cr_10_	183
	70%	Fe_30_Mn_50_Co_10_Cr_10_	38
		Fe_50_Mn_30_Co_10_Cr_10_	32
		Fe_80_Co_10_Cr_10_	108
	80%	Fe_30_Mn_50_Co_10_Cr_10_	18
		Fe_50_Mn_30_Co_10_Cr_10_	14
		Fe_80_Co_10_Cr_10_	57
Gyroid	60%	Fe_30_Mn_50_Co_10_Cr_10_	78
		Fe_50_Mn_30_Co_10_Cr_10_	66
		Fe_80_Co_10_Cr_10_	197
	70%	Fe_30_Mn_50_Co_10_Cr_10_	41
		Fe_50_Mn_30_Co_10_Cr_10_	33
		Fe_80_Co_10_Cr_10_	117
	80%	Fe_30_Mn_50_Co_10_Cr_10_	22
		Fe_50_Mn_30_Co_10_Cr_10_	17
		Fe_80_Co_10_Cr_10_	65
Sea urchin-inspired shell	70%	Fe_30_Mn_50_Co_10_Cr_10_	56
		Fe_50_Mn_30_Co_10_Cr_10_	49
		Fe_80_Co_10_Cr_10_	162
	80%	Fe_30_Mn_50_Co_10_Cr_10_	51
		Fe_50_Mn_30_Co_10_Cr_10_	39
		Fe_80_Co_10_Cr_10_	131
Bulk	0%	Fe_30_Mn_50_Co_10_Cr_10_	337
		Fe_50_Mn_30_Co_10_Cr_10_	302
		Fe_80_Co_10_Cr_10_	1042

## Data Availability

The original contributions presented in this study are included in the article. Further inquiries can be directed to the corresponding authors.
